# Computer Simulation of Magnetic Resonance Angiography Imaging: Model Description and Validation

**DOI:** 10.1371/journal.pone.0093689

**Published:** 2014-04-16

**Authors:** Artur Klepaczko, Piotr Szczypiński, Grzegorz Dwojakowski, Michał Strzelecki, Andrzej Materka

**Affiliations:** Medical Electronics Division, Institute of Electronics, Lodz University of Technology, Lodz, Poland; Universidad de Castilla-La Mancha, Spain

## Abstract

With the development of medical imaging modalities and image processing algorithms, there arises a need for methods of their comprehensive quantitative evaluation. In particular, this concerns the algorithms for vessel tracking and segmentation in magnetic resonance angiography images. The problem can be approached by using synthetic images, where true geometry of vessels is known. This paper presents a framework for computer modeling of MRA imaging and the results of its validation. A new model incorporates blood flow simulation within MR signal computation kernel. The proposed solution is unique, especially with respect to the interface between flow and image formation processes. Furthermore it utilizes the concept of particle tracing. The particles reflect the flow of fluid they are immersed in and they are assigned magnetization vectors with temporal evolution controlled by MR physics. Such an approach ensures flexibility as the designed simulator is able to reconstruct flow profiles of any type. The proposed model is validated in a series of experiments with physical and digital flow phantoms. The synthesized 3D images contain various features (including artifacts) characteristic for the time-of-flight protocol and exhibit remarkable correlation with the data acquired in a real MR scanner. The obtained results support the primary goal of the conducted research, i.e. establishing a reference technique for a quantified validation of MR angiography image processing algorithms.

## Introduction

Magnetic Resonance Angiography (MRA) is a powerful technique for visualization of blood vessels. Within the MRA setting, there exist both non-invasive imaging protocols, such as e.g. Time-Of-Flight (ToF) [1]–[3] or Phase Contrast Angiography (PCA) [4], and sequences that rely on application of contrast agent [5]. In this paper we focus on the ToF sequence.

Direct examination of the measured ToF images provides essentially qualitative description of the vessel system. Nonetheless, the quantitative information about the defects of vascularity of a visualized organ can help decide on the appropriate treatment. Such data can only be obtained with the use of specialized MRA image processing algorithms. If conducted properly, a quantitative analysis can increase objectivity and correctness of the medical diagnosis. For instance, the methods for vessels segmentation and tracking [6]–[9] enable an automatic identification of occlusions, pathological narrowings and hyper- or neovascularizations.

However, prior to the introduction of an image-based decision supporting system to clinical practice, it must be verified in a series of experiments involving a large number of test images, how reliable and accurate particular methods are. A statistically credible validation is difficult due to relatively high costs of collecting images solely for the research purposes. Additionally, MR scanners are extensively used in clinics and therefore are rarely accessible for experimentation. In consequence, the number of images available for evaluation studies is too low and there appears a need for a technique to generate synthetic images.

The problem of simulating the magnetic resonance imaging (MRI) has been studied over the last two decades and several computer simulators have been designed. The proposed solutions differ in the approach to model tissue of the imaged objects, in the routines of MR image synthesis, and in the degree to which different artifactual or undesired phenomena (like noise, chemical shift, ringing or Gibbs phenomenon, partial volume effect etc.) are taken into account.

In the approach proposed in [10], the imaged objects are defined as parameter maps. The maps visualize distribution of 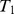 (spin-lattice relaxation time constant), 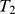 (spin-spin relaxation) and 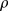 (proton density) values within the object. These values are calculated from real images. Formation of new images is performed using different repetition and echo times. On the other hand, in paper [11] the virtual organ object is modeled using the concept of spin density. By the use of inverse Fourier Transform, the simulator constructs the image representation in the spatial-frequency domain (k-space formalism). Simulation of the signal sampling phase and specific imaging sequence is accomplished through appropriate selection of the k-space elements and their amplitude and phase alteration. Separate simulation phases are required for each of the modeled tissues. This fact introduces a significant obstacle if a voxel contains more than one tissue leading to a partial volume effect.

In comparison with other approaches, more realistic simulation effects are achieved by solutions proposed in [12]–[15]. The models of the used imaged organs require that for each object voxel and every tissue component, relaxation times 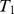 and 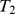, proton density 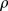, as well as value of the frequency offset linked to chemical shift artifact are defined. Consequently, to deal with the partial volume effect it is sufficient to determine the volume of particular tissue in a voxel.

It must be underlined that the simulators proposed so far can be used to synthesize only anatomical images, where the flow effects, suppressed by the imaging protocol parameters (echo and repetition times and RF pulse flip angle) are neglected. However, when it comes to MR angiography, beside nuclear phenomena, it is the motion of the hydrogen nuclei that produces the measured signal. Since none of the currently available simulators can be employed for MRA image synthesis, this paper presents an original computer system for simulation of the 3D ToF sequence.

## Methods

The design of the MRA simulator decomposes into the following tasks:

1. geometry definition of a vessel or a set of vessel branches (i.e. vascularity of an organ, hereafter shortly referred to as a *vascularity object*),
2. simulation of blood flow through the vessels,
3. modeling of stationary tissues which surround vessels (i.e. an *organ*),
4. simulation of magnetic resonance imaging sequences.

**Figure 1:**
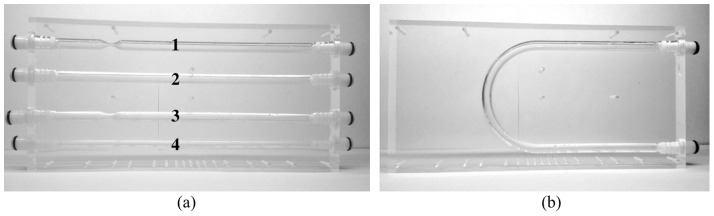
Flow phantoms used in the study. Channel diameters: 8 mm (1, 2, 3 and U-bend) and 5 mm (channel 4). Stenoses: channel 1 (75% of the diameter), channel 3 (50% of the diameter). Straight channels length: 207 mm. Curvature radius of the U-bend channel: 44 mm.

At first the blood flow and a vessel model is described. Since the aim of the research is a validation of the designed simulator, the focus is narrowed to relatively simple virtual phantoms of vascularity objects. Geometry of these models was adjusted to match real flow phantoms manufactured by Shelley Medical Imaging Technologies [16] (see Fig. 1) for the purpose of MR scanners calibration. This approach is motivated by the idea of reconstructing similar, corresponding to each other sets of images both in a simulator and a real MR scanner. The quantified degree of such correspondence enables an objective verification of the synthesized images from the point of view how realistic they are. The flow model description is succeeded by a presentation of the stationary tissue model and MR image formation procedures.

Eventually, details of real MRA image acquisitions are provided. Apart from the physical phantoms, the experimental apparatus consisted of the MR compatible, high-precision flow pump, CompuFlow 1000 MR, also manufactured by Shelley [17]. The pump is capable of forcing the flow at wide range of rates (from 0.1 ml/s to 35 ml/s) characteristic of human vessel system. It is also possible to generate a specific pulsatile flow waveforms, choosing either between one of the typical physiological courses (like common carotid or femoral waveforms) or a user-defined function. In the described experiments, however, only a steady flow option was used. It simplifies comparison of real and simulated images by removing additional factor causing potential discrepancies resulting from imperfect reproduction of the same pulsatile waveform in a flow simulation software. The pump is constructed mainly from non-ferromagnetic materials and is thus allowed to operate at high magnetic fields such as in the MR environment for the purpose of MR scanners calibration.

Furthermore, to achieve realistic conditions of the experiments, the medium used with the flow phantoms was Shelley's blood mimicking fluid (BMF). It is essentially a water solution of glycerol in proportion 6∶4. The physical properties of the fluid are presented in Table 1. All further computer simulations, with respect to flow and MR image formation, were performed using the same 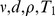, and 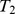 parameters.

**Table 1:** BMF physical parameters.

Parameter	Value
Density *d*	1020 kg/m^3^
Viscosity *ν*	4.1 mPa s
Proton density *ρ* (water relative)	1.00
*T*_1_ @ 1.5 T	850 ms
*T*_2_ @ 1.5 T	170 ms

### Blood flow and vessel model

A simulation of flow is performed in COMSOL Multiphysics software. The routines implemented therein solve Navier-Stokes equations for the incompressible fluid. A laminar flow, known also as stratified flow, assumes that fluid moves in parallel layers [18]. Each layer has its own speed and slides past one another with no lateral mixing. Blood flows in one direction with the parabolic velocity profile reaching the greatest value in the middle of the cylinder and it decreases towards vessel walls. The model is sufficiently accurate for simulating blood flow in a straight tube of relatively large diameter. However, in case of more complex structures with stenoses and bifurcations the flow behavior locally changes and turbulences and frictional forces should be taken into consideration [19]. Therefore, in the presented study the flow simulation is conducted in the turbulent regime using the RANS k-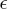 equation [20].

**Figure 2:**
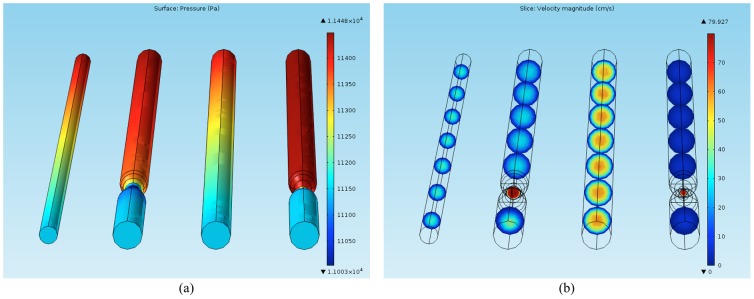
Blood flow simulation in the 4 straight tubes phantom. Simulated pressure (a) and velocity magnitude (b) distribution. Pressure difference (300 Pa) between channel inlets and outlets is made equal for every tube. Note the rapid increase in the velocity value and simultaneously pressure drop in the area of stenoses.

Among other available options, fluid motion in a vessel can be forced by determining pressure difference between the inlet and outlet surfaces. From the physiological point of view, the reasonable rates of peak velocity of blood for medium and large arteries(such as those modeled by the Shelley phantoms) range from 30 to 100 cm/s and the pressure values must be adjusted accordingly. Figure 2 presents a simulated distribution of pressure and velocity inside digitally reconstructed phantoms, where input and output pressure values were set to 11448 Pa and 11148 Pa respectively. Additionally, vessel walls are assumed solid, which prevents leakage.

**Figure 3:**
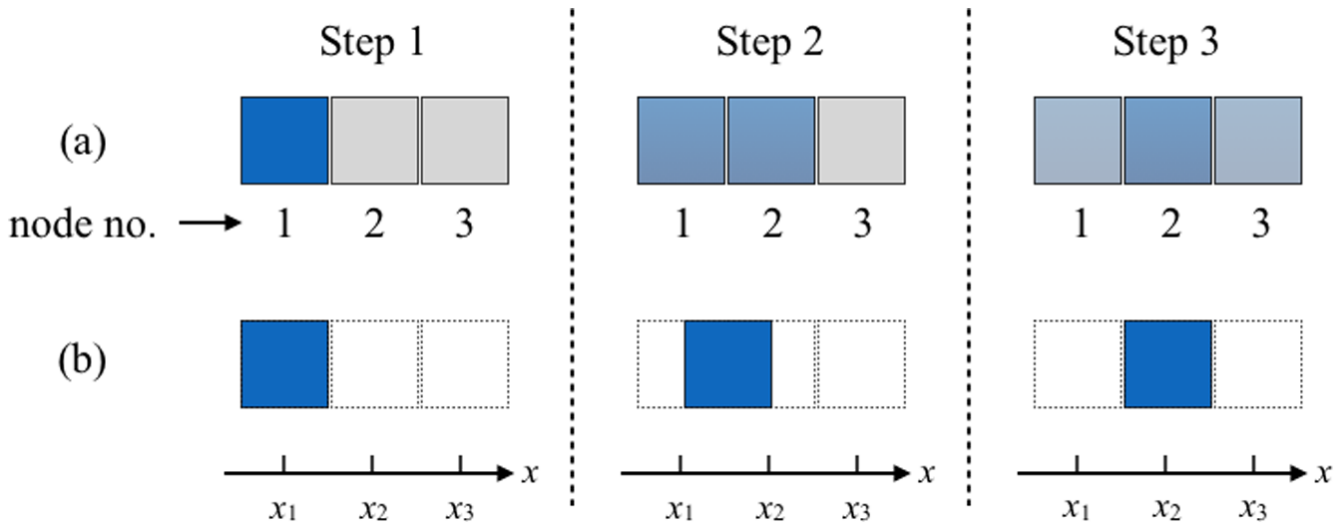
Illustration of the numerical diffusion problem. Fluid transportation in the case of a fixed mesh (a) and actual translation of a fluid portion along the 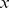 coordinate (b). In the former case, a fluid portion originally occupying node no. 1 spills over three consequtive nodes at time step 3 although in fact during this time period it has only and entirely moved from position 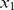 to 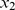.

It is important to note that COMSOL splits the domain of interest (in our case – the fluid) into a mesh of 3D geometrical primitives – tetrahedral nodes – of some finite volume. Velocity vectors are calculated for every mesh node. A crucial issue, is how to transfer this information in a usable form into the virtual MR scanner. A direct approach involves an operation on the COMSOL-generated mesh but it breeds a problem, referred to as numerical diffusion [21], [22]. An example of fluid motion in Fig. 3, showing positions of a portion of blood in 3 subsequent time steps illustrates the problem. If the flow channel is decomposed into a mesh of regular square-shaped nodes (in fact, any shape of mesh primitives entails similar consequences) it seems that in step 2, only half of the fluid volume shifts from node 1 to node 2. Thus, any further mesh-based calculations would require a division of fluid volume of this particular blood portion into 2 fractions, each for nodes 1 and 2. In step 3, these fractions move on: a part of blood residing in node 2 is transported to node 3, and again only a fraction of node 1 volume shifts towards node 2. Apparently, a sample of blood which initially occupied only one mesh node, spilled over 3 nodes. Clearly, this leads to an ambiguous localization of fluid portions – an error which accumulates in subsequent time steps. Moreover, this problem affects simulation of magnetic resonance, where subsequent image formation procedures (selective excitation, spatial encoding etc.) are invoked in discrete time moments and applied to portions of fluids at specific locations. Hence, a numerical diffusion may cause misinterpretation of true position of some blood samples.

**Figure 4:**
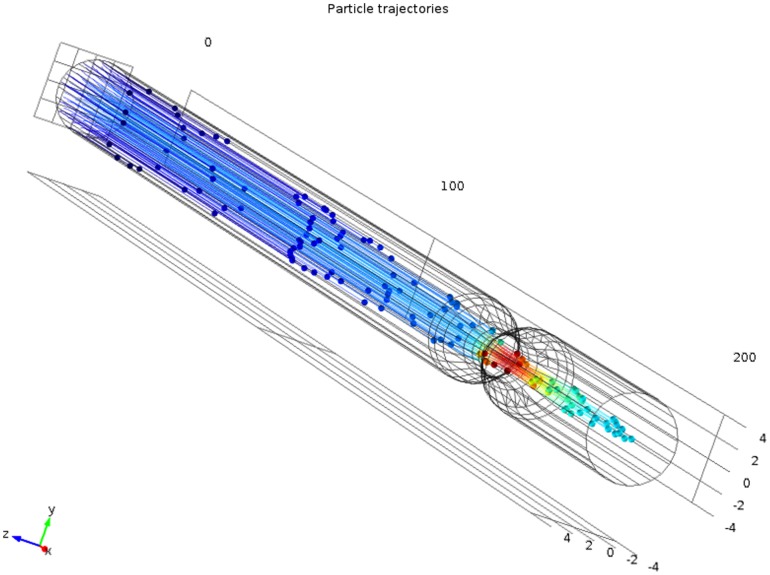
Particle trajectories as determined by the Particle Tracing Module. Simulated trajectories at a time step 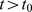, where 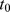 indicates initiation of flow. The fastest particles move close to the main axis of the tube.

To avoid the above described problem another COMSOL toolbox, namely the Particle Tracing for Fluid Flow module [23] is used. Based on the laminar and turbulent flow solution, the group of particles fed into a tube at an inlet is transported to its outlet. Each spherically-shaped particle is described by its density and diameter. Furthermore, the simulation is configured in such a manner, that there is no particle-fluid interaction. On the other hand, fluid forces particles to move along a vessel and by tracing their locations in subsequent time steps it is possible to find a bundle of trajectories which represent the motion of blood (see Fig. 4). These particle trajectories define the input to the MRA simulator.

**Figure 5:**
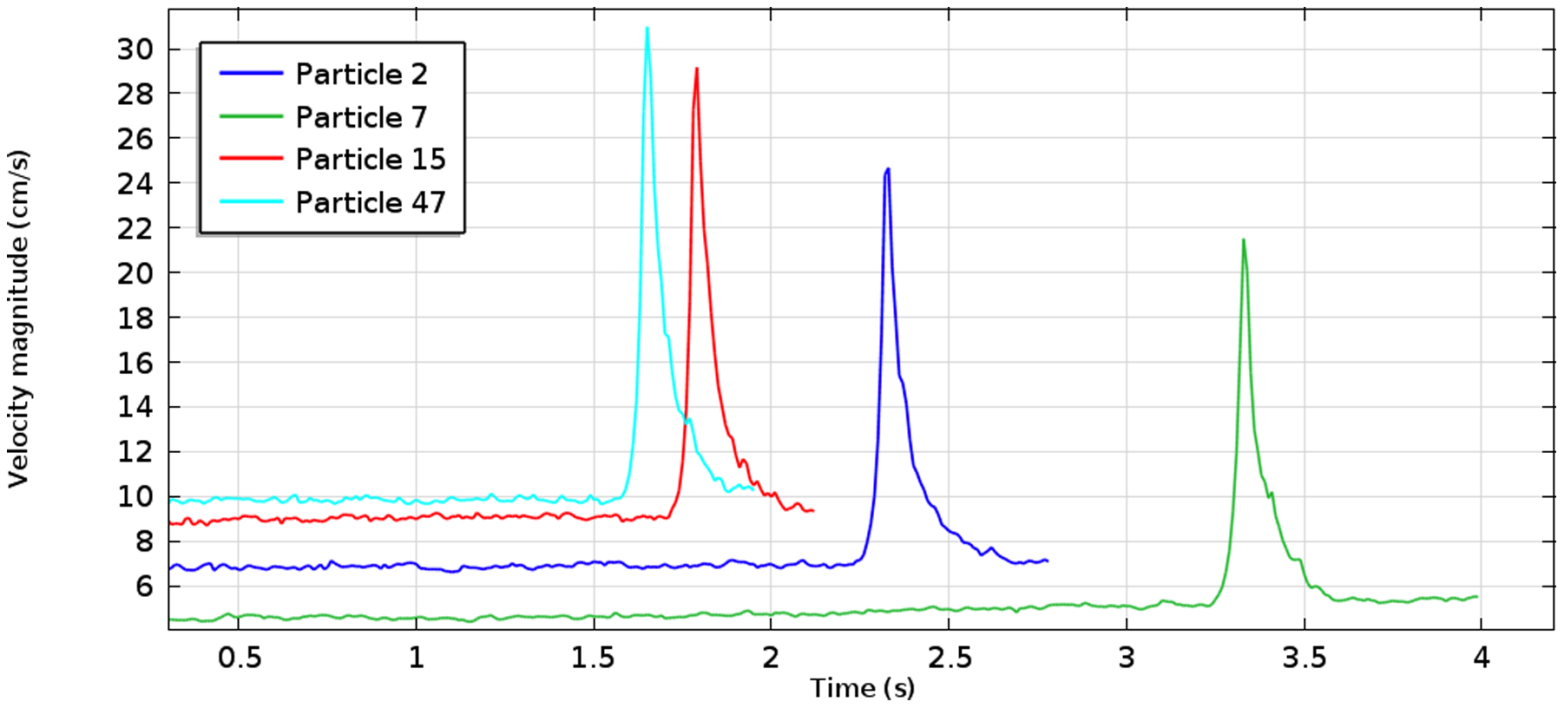
Time courses of velocity magnitude plotted for 4 randomly picked particles. Simulation performed for the 50%-stenosis tube with the flow rate of 2.5 ml/s. Particle which move faster (labeled 15 and 47) reach the narrowing earlier and gain larger peak velocity in the stenosis region than particles 2 and 7.

**Figure 6:**
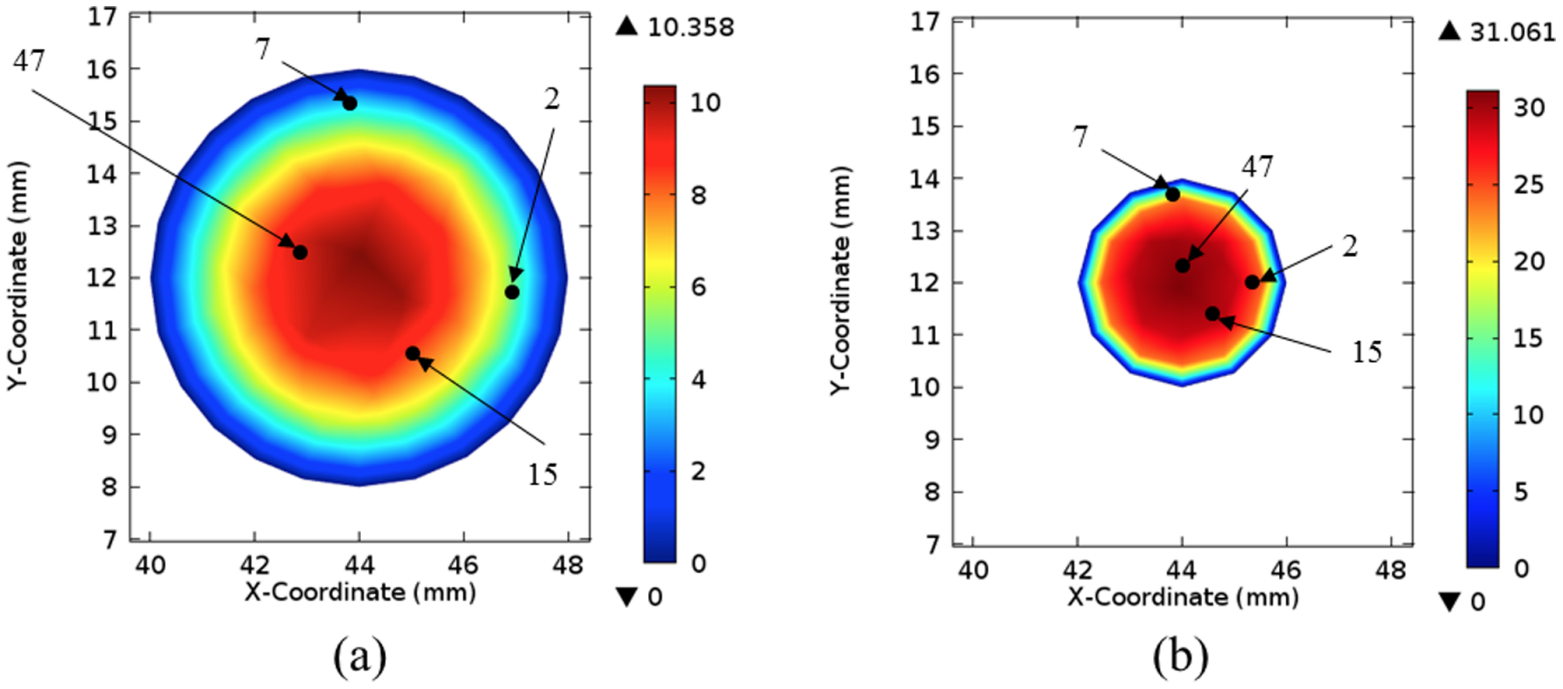
Distribution of velocity magnitude at two cross-sections of the 50%-stenosis tube [cm/s]. Fluid flow rate equals to 2.5/s. Black dots depict approximated locations of 4 particles whose velocity time courses are shown in Fig. 5 The selected cross-sections are situated a) 100 mm from the vessel inlet and b) in the middle of stenosis.

It is instructive to analyze the velocities achieved by the particles, as they move along a vessel, and compare them with the values obtained in a fluid flow simulation. Figure 5 presents time courses of the velocity magnitude plotted for four randomly picked particles in the experiment inside the 50%-stenosis tube. It has been observed that the velocities remain constant until particles reach the narrowing, where they experience rapid acceleration, then they slow down and finally flow out from the vessel. The initial velocity value and the moment of its increase differ between the particles, depending on their position relative to central axis of the channel. Figures 6a–b depict locations of the same four particles in two cross-sections of the vessel situated either at 100 mm from the inlet or in the middle of stenosis. Also, velocity magnitudes, as calculated by the fluid flow simulation, are shown. Clearly, particle velocities presented in the time courses remain close to the values obtained using the plots in Fig. 6. It demonstrates the equivalence between a standard flow simulation and particle traces.

### MRA Imaging Simulation

**Figure 7:**
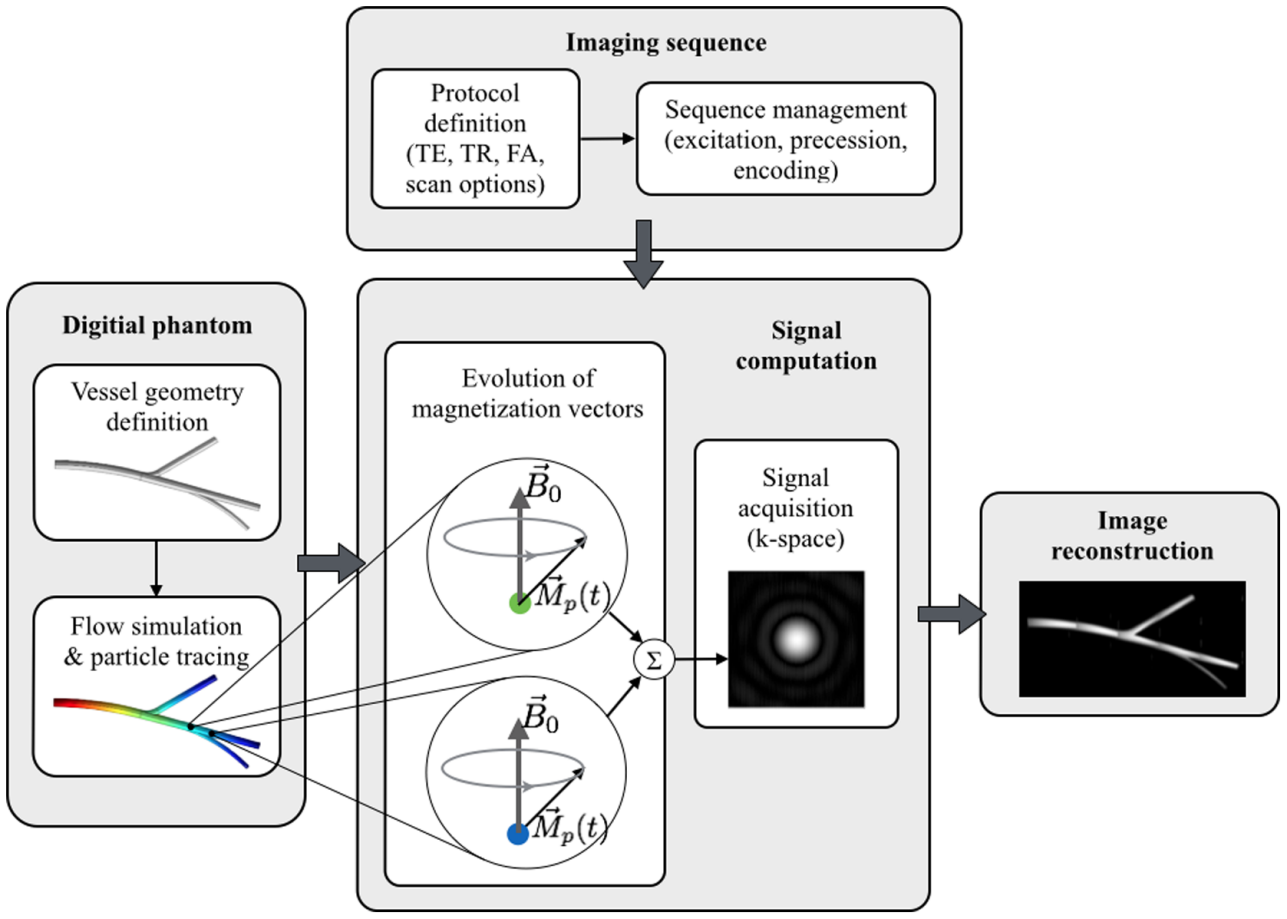
Main modules of the designed MRA simulator. The simulator is decomposed into 4 integral parts: 1) virtual object description, 2) sequence management, 3) signal computation and 4) image reconstruction.

The main software modules of the designed simulator are presented in Fig. 7 and described below.

#### Digital phantom

The input to the MRA simulator is a model composed of particle trajectories as described above. These trajectories are defined by a series of particle coordinates at subsequent flow simulation time steps 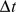. In fact, the coordinates can be easily interpolated in time domain, which in turn allows the selection of any other time step for the purpose of the subsequent stages of the simulation. In other words, in both stages i.e. in the simulation of flow and in the simulation of magnetic resonance effects, the discretization of time domain can be adjusted independently.

In the MRA simulation, the whole volume of a vascularity object has to be filled with particles. Furthermore, every particle corresponds to a portion of the fluid so that the sum of all these portions constitute the total volume of the blood flowing through the vessels. Therefore, in the proposed method every trajectory is populated by particles according to the following strategy.

**Figure 8:**
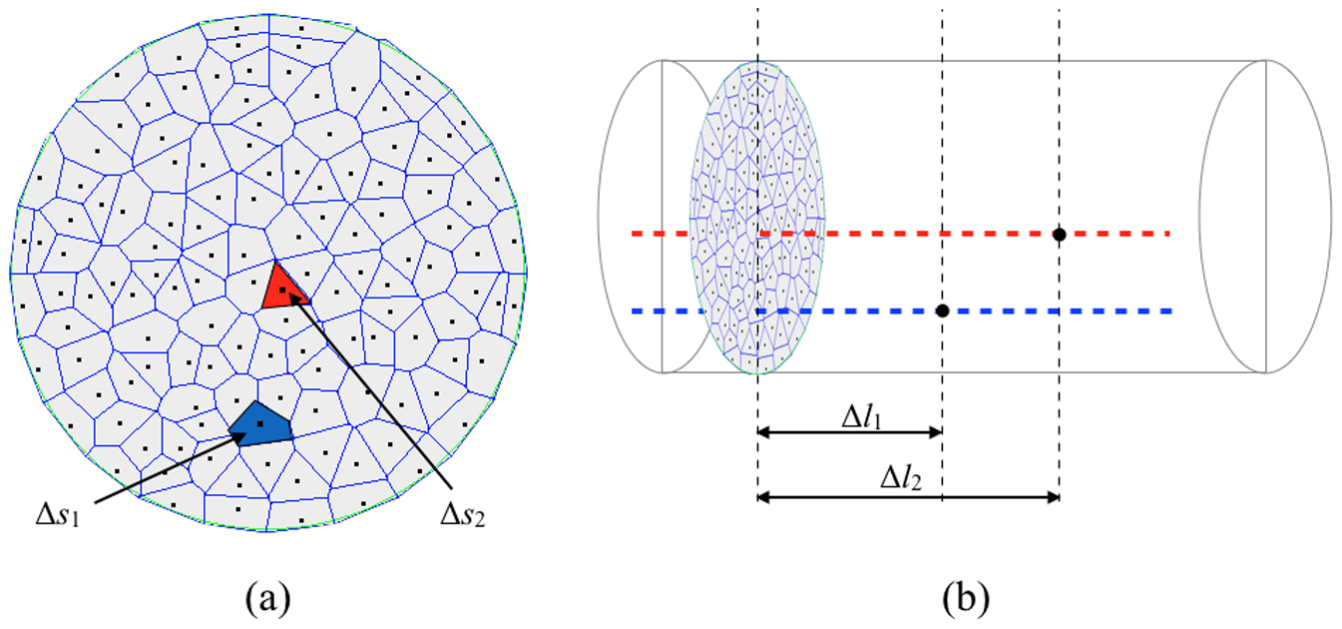
Calculation of particles cell volume. a) The result of Voronoi tessellation of a tube cross-section. Particles (black dots) cast on the selected surface serve as seed points. The blue and red polygons are example base faces of particle-associated cells. b) Lengths 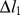 and 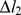 denote distances traveled by particles on trajectories 1 (blue line) and 2 (red line), both during the same time interval 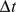. 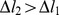 as velocity on the red trajectory is larger.

Firstly, the area of a tube inlet, which should be perpendicular to the course of trajectories, is tessellated by means of the Voronoi decomposition (Fig. 8a). It divides the inlet area into regions, so that every region corresponds with the individual trajectory. As a result, every trajectory is assigned an area 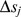, where the 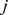 indicates a particular trajectory. In consequence, contributions from trajectories sparsely scattered are balanced with respect to those which are distributed more densely. Secondly, let us consider a right prism in which an individual Voronoi cell constitutes a base polygon and the distance between the two base polygons is equal to 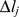 (Fig. 8b). The volume of such prism is a product of the base face area 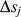 and the distance 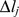. Therefore, in case of a straight tube, i in which the fluid speed along the trajectory is stable and the tube's cross-section remains constant, the trajectory is populated with particles equally spaced by the distance 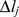. The volume associated with every such particle is equal to the volume of the right prism mentioned above.

As the MRA simulation advances, the particles move along the associated trajectories toward the tube outlets and finally, as they pass through the outlet, they disappear from the simulation. At the same time new particles must be introduced at the inlet of the tube, as the vascularity object should be filled with particles at all times of the simulation. The new particles are introduced at a specific time rate 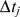 (not to be confused with the flow simulation time step 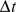) equal to the ratio of the distance 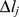 and speed of the fluid along the corresponding trajectory. Therefore, every particle introduced at the inlet is distant from the preceding one by the 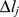 and represents the same amount of the volume equal to 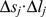. The 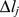 and consequently 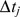 can be adjusted individually for each trajectory. In our approach, by default they are chosen so that the distances between adjacent particles on the same trajectory are comparable to the distances between the neighboring trajectories, giving an even distribution of particles in all three dimensions. These values however, can be manipulated to achieve a desired number of particles along trajectories.

Note that in a narrowing particles move faster and the distance 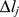 becomes larger. At the same time the area of vessel cross-section shrinks, 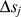 decreases with inverse proportion to the increase of 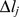 and as a result the volume corresponding with the particle remains constant.As the particles on different trajectories convey various portions of blood, the associated volume is used to weight the MR signal originating from its corresponding particle. It is important that information on exact shape of boundaries limiting the volume is not further required. In consequence, there is no need for exact tessellation of the three dimensional volume of the vascularity object, which in turn simplifies the simulator implementation.

As indicated above, the time step of MRA simulation 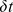 generally differs from the step used in flow simulation 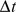 and also from the steps 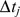 used to fill trajectories with particles. The system finds position of a particle at time 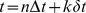 by interpolating its coordinates corresponding to two locations, one at time 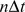 and the other at 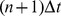, where 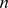 and 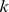 are respectively the flow and MRA simulation step numbers. This solution enables performing image synthesis at arbitrary temporal resolution.

Apart from volume and parameters related to their motion, particles are assigned 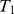 and 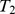 relaxation times and proton density value 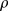. Eventually, each particle on a trajectory obtains a unique identification label allowing to trace it during image formation. A description of the digital phantom is completed by the information about physical size of an imaged organ and position of the vessels within it.

In the performed experiments the number of trajectories in a single tube was set to 256. Furthermore, the particle-associated volumes were adjusted to achieve the ratio of 3 particles per millimeter of a vessel length. It gives the value of about 20 particles per cubic millimeter or about 12 particles per final voxel. We found this ratio as optimal to obtain realistic images, while avoiding computational overload. In lower resolution images, also the number of trajectories may be decreased.

Stationary tissue which surrounds vessels – for clarity not shown in Fig. 7 – is modeled by a set of particles similarly to flowing spins but a position of the stationary particle is fixed for the entire scope of simulation. The definition of the stationary tissue requires setting of the shape and size of the organ. Then, the number of stationary particles is adjusted to match the density of their distribution to the ratio obtained for the moving ones. Tissue particles are uniformly distributed within the imaged volume except for the regions occupied by vessel branches. Furthermore, particles are assigned their 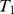, 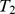 and 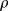 values. Eventually, there can be several tissue types present in the imaging volume. Every component is simulated separately, and the final image is reconstructed from the sum of signals acquired for each tissue. Moreover, the chemical shift between fat and water components can be defined to simulate spatial misregistration of fat protons caused by magnetic shielding of the electron shell altering their effective resonant frequency. Nevertheless, the effect of chemical shift is not considered in the experiments reported in this study.

#### Imaging sequence

*Protocol definition.* An image synthesis procedure is controlled by a group of parameters constituting a sequence type and output image contents. The latter refers both to the image resolution (k-space size) and to the position and size of the field of view (FOV). By default, FOV embraces a whole object, but it can be reduced to only a part of it and its center can be shifted to any object point. It is important to keep a record of the FOV center offset and its location relative to gradients isocenter, since it directly affects the field strength value experienced by each particle.

The sequence definition is mainly determined by the sequence type. The Time-of-Flight protocol utilizes the Spoiled Gradient Echo sequence (SPGR) [2]. The SPGR sequence leads to saturation of the stationary spins which in turn produce much lower signal than fresh unsaturated molecules flowing into the imaging volume. The degree of inflow enhancement depends on velocity of blood spins and the orientation of vessels relative to the acquisition slice.

As for MR experiment, the program requires a specification of the echo and repetition times (TE and TR accordingly) and also the flip angle (FA). The sequence type also includes information whether the target image is 2- or 3-dimensional. For the 2D image the slice position and orientation should be specified. In case of volumetric acquisitions it must be also defined whether the whole FOV is scanned as one thick volume, or as a series of thin slabs (MOTSA – multiple overlapping thin slabs acquisition technique) [24]. Moreover, for 3D ToF it must be determined whether the ramped RF pulses, with spatially varying ip angle parallel to the direction of ow, are to be used. This technique, sometimes called TONE (tilted optimized non-saturating excitation) [25], [26], reduces the effect of signal suppression from molecules which have traversed long distances within a slab and thus have been saturated by multiple RF pulses. Finally, the signal sampling window is determined by the readout time parameter which must ensure that the resulting sampling frequency meets the Nyquist-Shanon theorem.

*Sequence management.* An event management module is responsible for invoking subsequent steps of computations based on the current simulation stage. It begins with establishing the initial magnetization of particle spins by allowing them to freely precess in the presence of the main external magnetic field 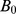. This procedure is later repeated for any new particles replacing those which flow out from vessel outlets. Then, the particles magnetization states are altered by an application of an RF pulse, free precession, and an application of phase encoding gradients. As for volumetric acquisitions and an in-plane flow in 2D, flow compensation gradients are included in the sequence design. These gradients account for a phase dispersion effect, taking place if spins of different speed flow through a voxel during the signal acquisition. Such spins have different phase and cause the signal from the voxel to fade away. By applying additional compensation gradients along the direction of flow, it is possible to partially eliminate this unwelcome effect.

After the phase encoding and – optional – velocity compensation steps, the frequency encoding gradient is applied and the signal acquisition step is executed. Eventually, numerical spoiling of the transverse magnetization is accomplished. At each step the MRI kernel is invoked with the appropriate parameters.

#### Signal computation

*Evolution of the magnetization vector.* The core of the system routines implements the discrete time analytic solution to the Bloch equation [27]. This approach relies on the assumptions of constant field 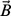 and that the shape of an RF excitation pulse is rectangular or it can be approximated by the set of such pulses. Thus, the simulation is limited to time-invariant gradient fields during excitation. In the generic case of arbitrarily shaped RF pulses, no analytical solution exists and numerical approach is required.

The analytical solution uses the rotation matrices and exponential scaling to modify magnetization vector of a particle (either moving or stationary) in accordance to specified sequence events. Hence, the magnetization vector 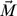 of a particle 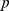 is given by
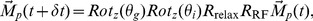
(1)where 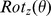 rotates the magnetization vector around the 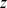-axis in reply to the phase encoding gradient (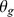) and as a consequence of the main magnetic field inhomogeneity (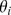). Note, that for the proper evaluation of the Bloch equation, position 
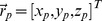
 of a particle relative to gradient isocenter must be determined first. For a stationary component, each particle has constant coordinates. In contrast, a moving particle changes position in time. Thus, a magnetization vector must be updated at time intervals which ensure reasonable trade-off between precision of the flow simulation, MR modeling and computational burden. Too high values of t introduce unacceptable discontinuity in evolution of the effective Larmor frequency experienced by a particle. On the other hand, very short 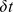 results in significant increase in simulation time. In our approach, 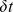 is adjusted to match sampling frequency of the signal acquisition phase, i.e. it is set to sampling window duration divided by the number of k-space points in the frequency encoding direction.

The 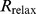 rotation matrix is responsible for the relaxation effects and is calculated as
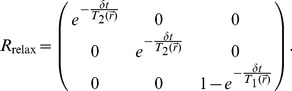
(2)The last term in (1) is the 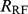 operator. In the general case, where the local field differs from 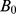 due to field inhomogeneity or chemical shift artifact, the resonance frequency 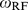 deviates from 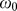 implied by 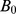. Hence, the effective precessional frequency is
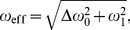
(3)where 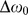 measures the frequency offset. Consequently, a 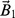 pulse of duration 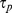 which is assumed to knock over 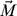 by angle 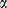 from 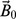 yields the effective flip angle
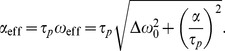
(4)In the off-resonance condition spins experience not only the 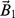 pulse directed (assuming rotating frame of reference) along the 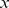 axis, optionally with a phase angle 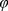, but also residual field 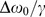 – where 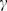 is gyromagnetic ration – which points along 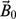. Therefore, the effective 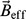 field forms an angle 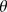 relative to the main magnetic field direction. Hence, the 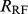 is eventually determined by
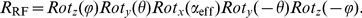
(5)


It is important to note, that 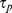 has to be divided into time intervals 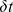 as in the other simulation phases (relaxation, gradient application). The blood particles move during RF pulse application and may not acquire sufficient energy to get excited. Thus, at each 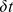 step the particle magnetization vectors are flipped only partially by angle 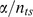, where 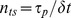. Moreover, in the case of TONE protocol, the equation (4) resolves to
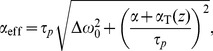
(6)where 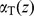 is the extra flip angle dependent on a particle 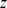-position and which adds to the base angle set for the entry slice.

*Signal acquisition.* A signal acquisition phase fills one line of k-space per TR. A single data point is calculated through numerical integration of transverse components of particle magnetization vectors over the whole imaging volume according to equation
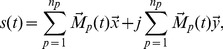
(7)where 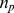 denotes number of particles.

Sampling frequency is determined by user-defined length of the sampling time frame and the desired number of voxels in the frequency encoding direction. Upon completion of one k-space point acquisition the system is allowed to alter its state - due to blood flow on one side, and relaxation phenomena on the other. After updating particles position and their magnetization vectors, next data point is calculated.

Note that because blood particles are subjected to flow, some of them may leave a vessel between two subsequent signal measurement times. New particles replacing those that have disappeared have no transverse magnetization as they did not receive any RF excitation pulse. As a result, they do not produce any signal until next echo cycle. This shows up in the resulting image as dark voxels in the slices where blood particles of high velocity enter imaging volume.

#### Image reconstruction

The last stage of image formation procedure consists in transformation of the raw data in the spatial-frequency domain (K-space) into image intensity domain. This stage is accomplished by the fast Fourier transform, followed by calculation of the magnitude of – complex transformation output. Before application of the FFT routine, it may be necessary to filter the raw data to reduce the phase mismapping artifact appearing in the phase encoding direction due to the motion of blood particles. We decided to port the filtering routine and FFT-based image reconstruction to the Matlab environment to enable any kind of low-pass digital filter available in the Signal Processing Toolbox [28] to be used.

#### Implementation

Apart from the image reconstruction module, the designed system has been implemented as a custom computer program. The source code was written in the ANSI-C language and compiled for the Mac OS X 10.7 platform (the OS X-native *clang* compiler was used). Moreover, the program was parallelized using Open MPI library [29]. The algorithm execution is divided into a set of computing agent-nodes, each responsible for handling a given bunch of trajectories. After calculation of the MR signal from its assigned trajectories, an agent sends the results to the master node who adds the received data to the signals simulated by other agents. If there are trajectories still waiting for assignment, an agent who completed its job is employed to handle another portion of data. This procedure ensures high efficiency as more powerful processing resources can be utilized more often. In our experiments the system was installed on the computer grid composed of one iMac desktop (Intel Core i7 3.4 GHz) and 10 MacMini units (10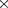 Intel Core i5 2.5 GHz) which is equivalent to 24 CPU cores. Additionally, if hyperthreading feature is taken into account, the execution can be distributed among 48 processing slots. The computers were connected over the local ethernet network and communicated on the SSH layer. The details of the SSH- and Open MPI-based grid setup are outlined in [30], where a heterogeneous computing environment extended on a Linux and legacy OS X (version 10.5) server is employed.

### Measurements

Image acquisition was performed on the GE Signa HDxt 1.5 T system for various flow rates and sequence parameters. In every case, the 3D images were acquired using MOTSA and TONE techniques, and with flow compensation option switched on in each dimension. Tables 2 and 3 summarize the experimental setup. Note that in TONE technique, the FA parameter is the nominal flip angle set in the middle of the slab and it linearly decreases or increases along the 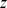 coordinate. On the other hand, simulation experiments are restricted to the two tubes with stenoses and the U-bend phantom, as their geometry seems to be more explanatory in quantitative inference.

**Table 2:** Flow rates and corresponding image acquisition parameters.

Study #	Flow rate [ml/s]	TE/TR/FA [ms/ms/–]	Slab width/slab overlap [mm]	FOV (x/y/z) [cm]
1	2.5	4.7/40/20°	44/8	17.5/13.4/14.4
2	2.5	4.7/40/15°	44/8	17.5/13.4/14.4
3	4.6	4.6/27/15°	44/8	17.5/13.4/22.4
4	10	4.6/27/15°	25/5	17.5/13.4/24.5

**Table 3:** Common image acquisition parameters.

Parameter	Value
Magnetic field strength	1.5 T
Protocol	Brain Vascular
Coil	1-channel transmit/receive bird-cage head coil
Scanning sequence	SPGR
Scanning options	Flow compensation, Variable flip angle (TONE), Spatial saturation, MOTSA
Slice thinckness	1 mm

**Figure 9:**
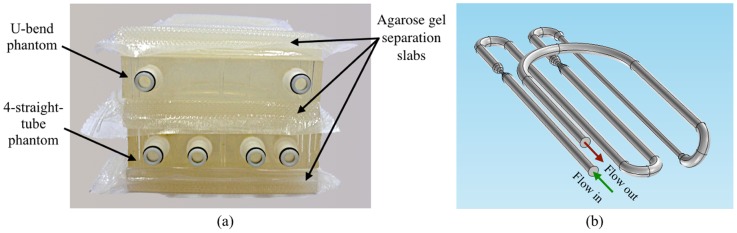
Assembly of the physical phantom set. (a) The stack of flow phantoms and separating agar gel layers. (b) Schematic of flow channel connections.

In addition to flow phantoms, the experimental set is completed by three agarose gel slabs which are placed within the magnet together with the phantoms, as illustrated in Fig. 9a. The purpose of these layers is to increase load for the RF coils and to enhance signal quality. Also they can be used to estimate noise level and perform background correction.

Both phantoms were simultaneously put into the scanner and the phantoms were interconnected as viewed schematically in Fig. 9b. In this setting, the fluid first enters the 75%-stenosis tube, then returns through the U-bend phantom to the pump. The phantoms inside the magnet bore were oriented in such a manner that the flow channels were placed parallel to the z axis i.e. parallel to the main magnetic field, and vertically to the transverse plane, thus maximizing the effect of inflow enhancement. In the transverse plane, the data space was sampled with 224 and 160 of respectively frequency and phase encoding steps. The measured k-space slices were then null padded to 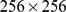
–pixel images. A field of view size was adjusted to entirely cover a cross-sectional area of the phantom set, while slabs depth, their number and consequently FOV along the 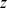 axis varied depending on the study (cf. Table 2). Eventually, the imaging protocol included spatial presaturation option. In such a protocol spins flowing against the direction of 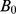 field enter a saturation band located above an imaging slab. They receive additional RF pulses and are saturated, thus giving no or a reduced signal during acquisition. The goal of this action is to separate signals of arterial and venous origin.

## Results

**Figure 10:**
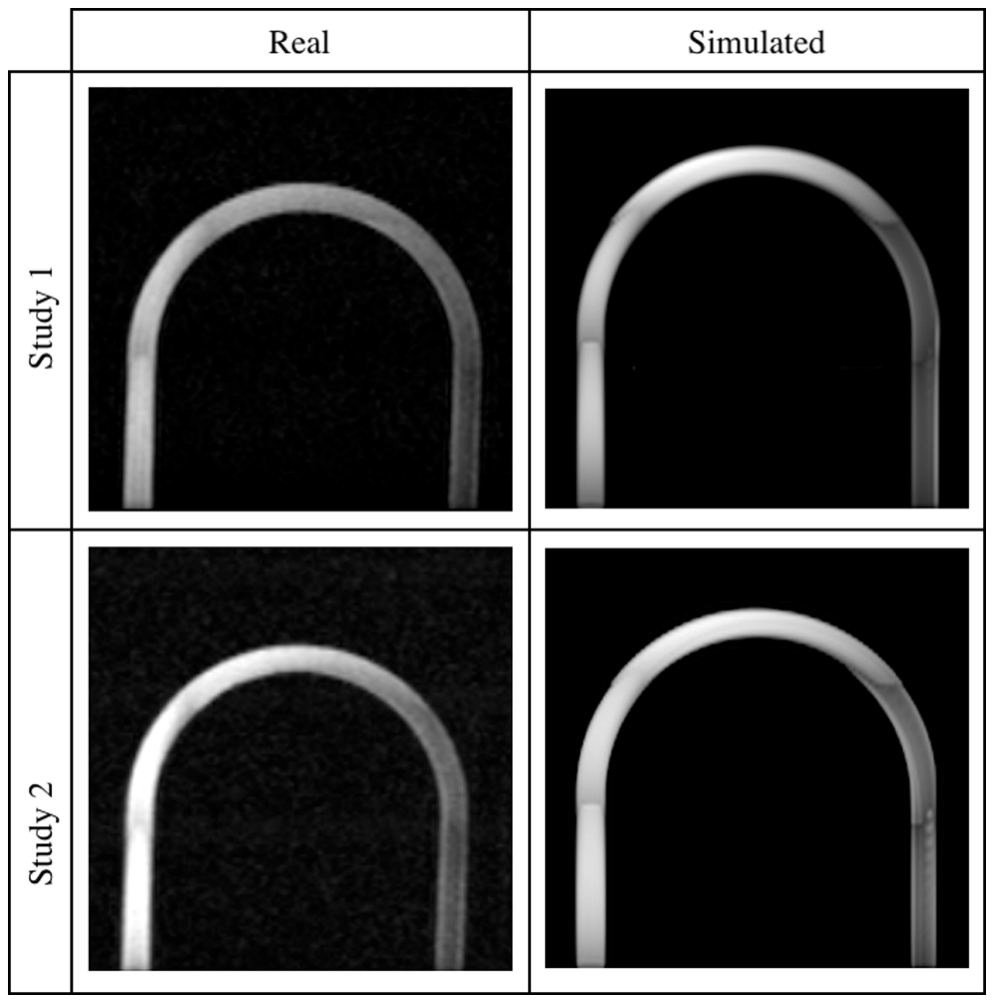
U-bend phantom images – Studies 1 and 2. Real (left panel) and simulated (right panel) acquisitions.

**Figure 11:**
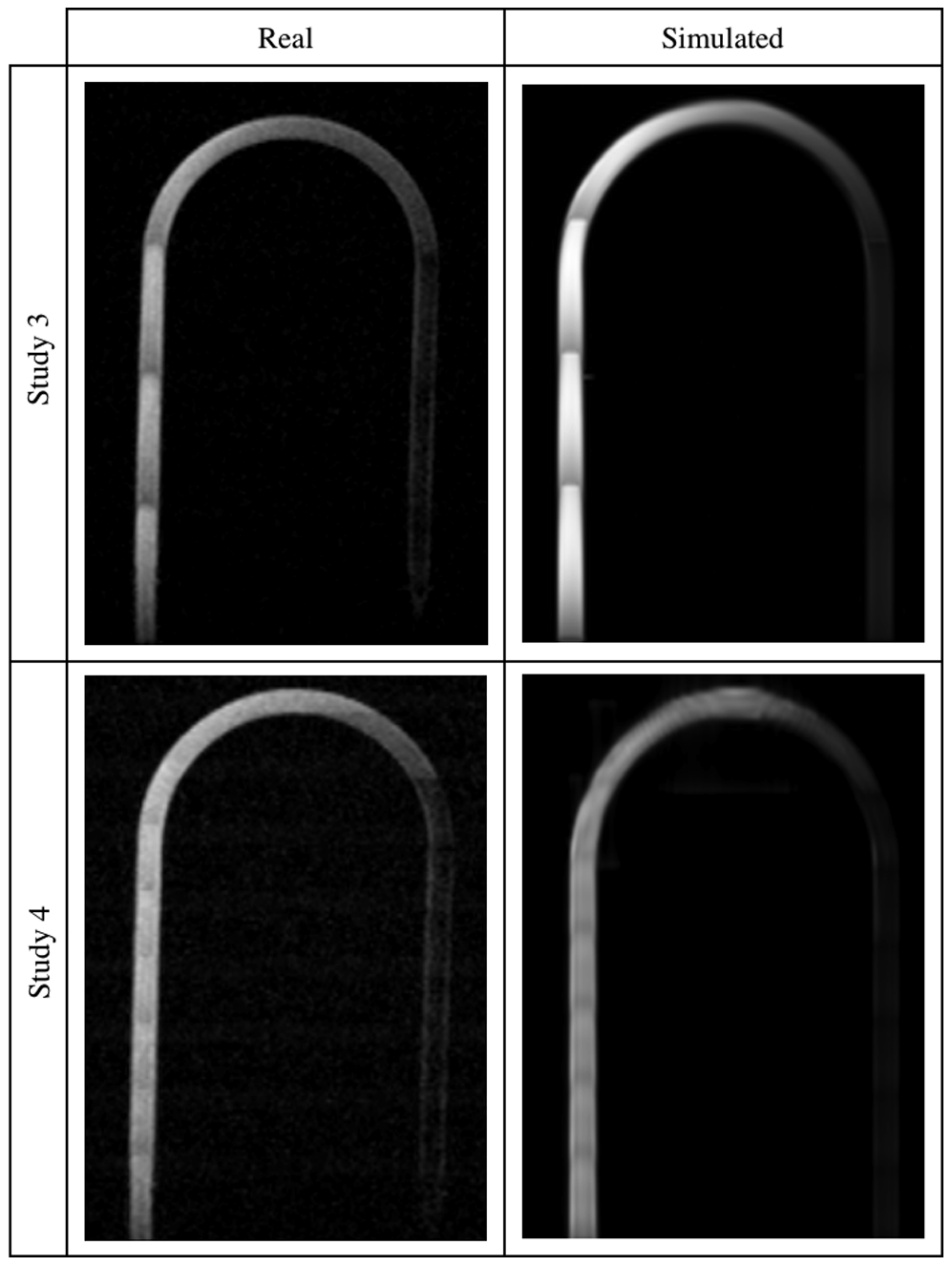
U-bend phantom images – Studies 3 and 4. Real (left panel) and simulated (right panel) acquisitions.

**Figure 12:**
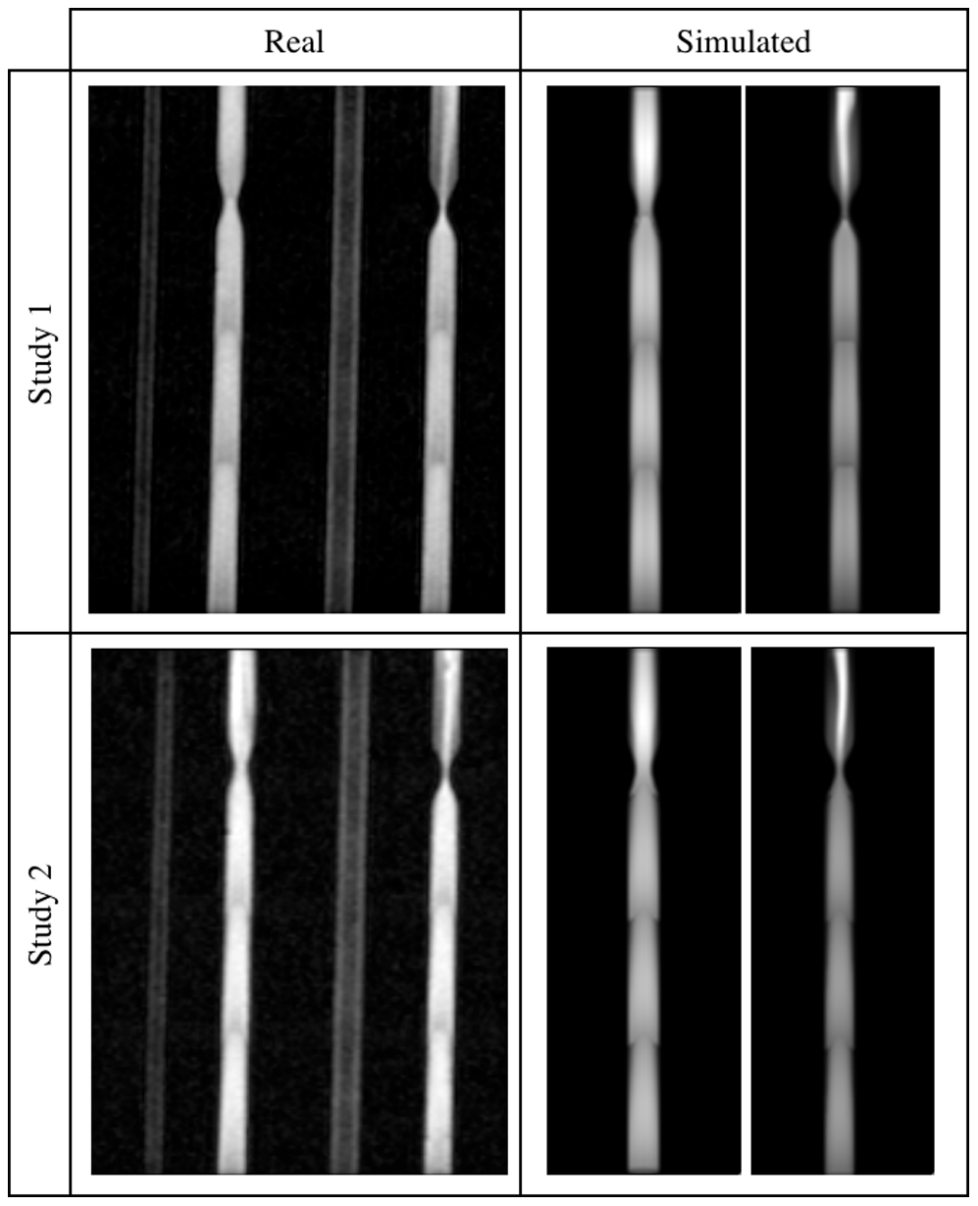
Straight tubes phantom images – Studies 1 and 2. Real (left panel) and simulated (right panel) acquisitions.

**Figure 13:**
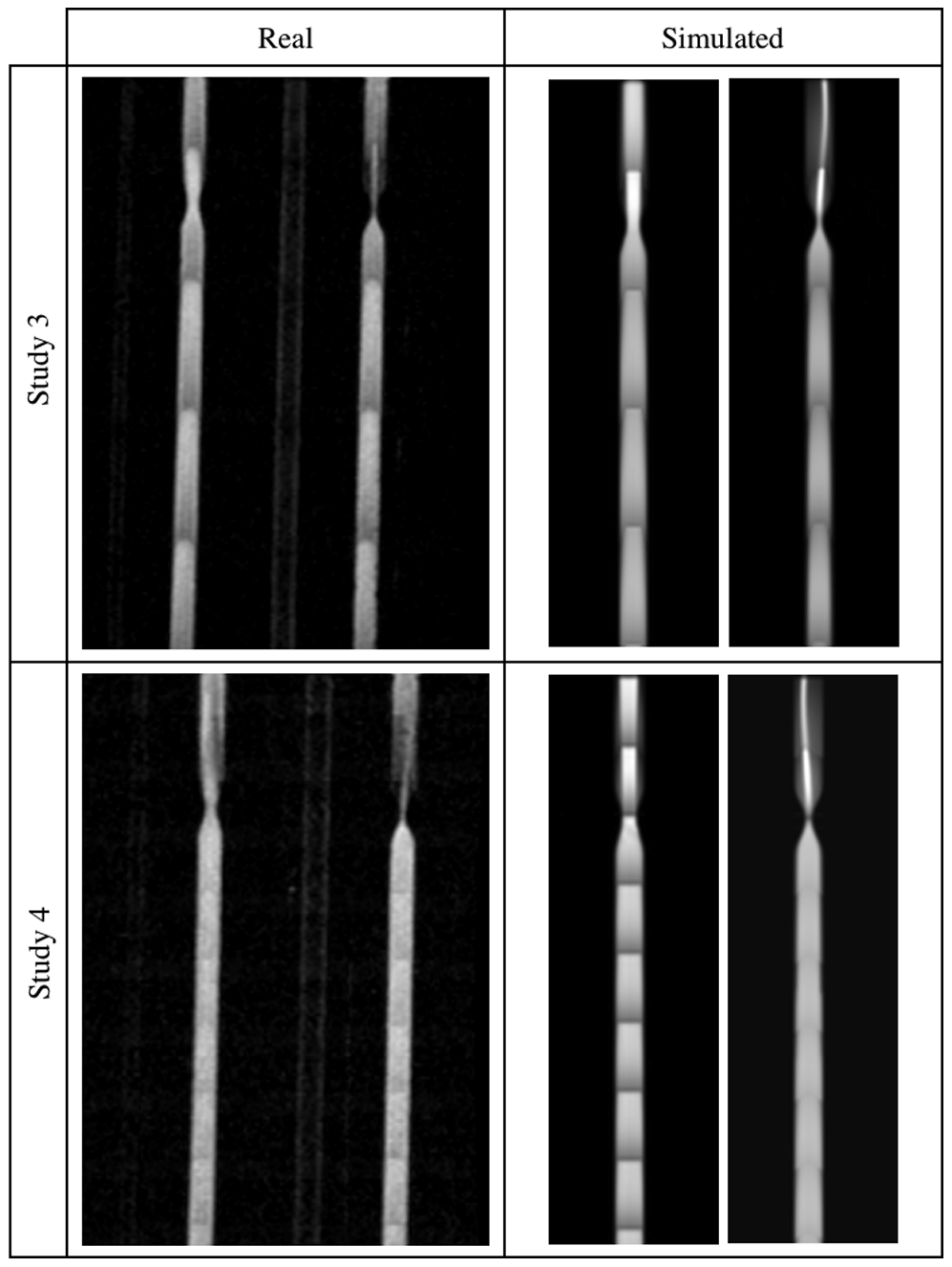
Straight tubes phantom images – Studies 3 and 4. Real (left panel) and simulated (right panel) acquisitions.

In order to validate the designed simulator system, a series of experiments using the above described physical phantoms imaged in a real MR scanner, was performed. On the other hand we synthesized equivalent images using our simulation framework forcing the same flow and imaging sequence parameters. A comparison of these two groups of images – real and synthetic ones – allows a comprehensive verification of correct implementation of the designed system. Figures 10–13 present the obtained real and their corresponding synthesized images.

### Simulation results – qualitative analysis

A visual inspection of the results reveals that the highest degree of similarity between these two sorts of data has been obtained for the lower and medium flow rates. First of all, in both types of images a characteristic signal void near the entry layers of each slab is observed. This effect is caused by the spins flowing faster which are flushed out from the area of their initial excitation and produce a signal deeper in the slab. Slower spins receive more RF pulses and become saturated before reaching the end of the slab. Consequently, image brightness decreases more quickly near the channel walls, when compared to its central tracts, giving a parabolic profile of the image intensity.

Next, it can be noticed that a signal from the return section of the U-bend phantom is low relative to the inlet channel. In this case the mechanism responsible for signal suppression is spatial presaturation. Apparently, the signal close to channel borders is brighter than that observed in the middle. This can be explained by the fact, that it takes same time for spins to travel from the saturation band to an imaging slab. Those spins which move slower i.e. near channel borders, have time to recover some portion of their longitudinal magnetization and thus produce a measurable signal. This effect is better visible in the real images, however still observable in the synthetic ones.

Moreover, image intensity at locations where the fluid only exits, immediately after passing a narrowing and close to central axis rather than channel walls, is significantly larger. It is especially manifested in the 75%-stenosis tube. The main flux – as it can be judged from the brightest voxels bundle – visible after stenosis is asymmetric relative to the tube axis. This fact is reflected in the synthesized images, although the deviation from the symmetry axis is slightly different. It seems to be a result of complex and chaotic flow patterns which appear after a narrowing. Hence, the need to take into account both laminar and turbulent models in the simulation of flow, becomes evident.

Also, one can compare images acquired for the same – 2.5 ml/s – flow rate, but for different nominal flip angle (i.e. 15° or 20°), see Study 1 and 2. As to a larger flip angle, the wash out effect on slab entries is reduced and also channels with reverse direction of flow (i.e. against 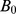 field) produce slightly higher signal in the channel lumen (observed for the U-bend phantom – Fig. 10). The same effects can be observed for the synthesized images.

On the other hand, images synthesized in Study 4 (with flow rate of 10 ml/s) appear less realistic. For example, in the case of the 50%-stenosis tube simulation intensity jumps between neighboring slabs are clearly visible, although in the real image they can hardly be noticed.

### Simulation results – quantitative analysis

#### Validation methodology

**Figure 14:**
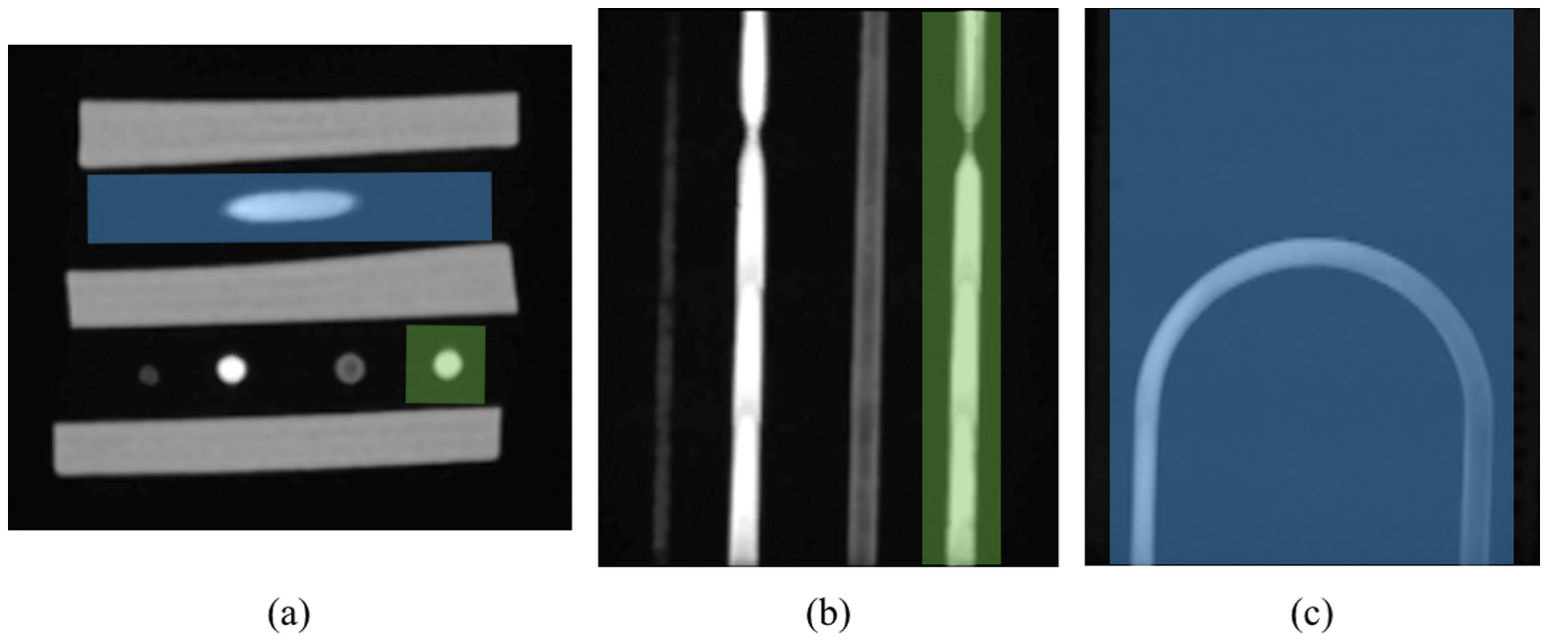
Examples of selected volumes of interest. Axial (a) and coronal (b and c) cross-sections of delineated VOIs embracing U-bend phantom (blue region) and 75%-stenosis tube (green region). Note that it is only a conceptual figure – the actual VOI selection was performed automatically in the MATLAB environment.

In the analysis described in this section we aim at comparing two sets of images – real and synthesized – objectively, i.e. in the quantitative manner. In order to accomplish this task we propose the following methodology. First, we select volumes of interest (VOIs) in each examined 3D image, which contain isolated tubes with stenosis and the U-bend phantom. Figure 14 shows outline of two exemplar VOIs selected in real images for the 75%-stenosis and the U-bend tubes. VOIs selection was performed manually simply by appropriate indexing of the image data loaded to the MATLAB workspace. As for the tubes with stenoses, the size of the subvolumes are 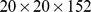 voxels, while the U-bend phantom was cropped to a region of size 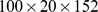 voxels. The determined VOI depth ensures that a vessel is embraced at its entire length, while width and height are adjusted to account for oblique vessels orientation. The observed distance range between all voxels from a single tube projected onto an axial plane does not exceed the value of 20 points. Similar VOIs were selected both in the real and synthetic images.

Note that the flow channel axes in the real images are neither perfectly parallel to the z axis of the image space coordinate system, nor vertical to the transverse plane. Thus, in order to compare them with the simulation results on the voxel-by-voxel basis, the real and synthetic images must be registered. We perform this procedure within respective VOIs using MATLAB Image Processing Toolbox [31]. The employed algorithm matches two volumetric data sets by performing rigid transformation of a synthesized image and optimizing their mutual information. Having registered VOIs, we calculate correlation coefficients and also – after scaling intensity values to span the same range in both data sets – root mean squared errors between corresponding images.

#### Image denoising

It must be noted that real images contain noise component, not included in the synthesized images. Noise degrades quality of images which hampers their visual examination and affects their quantitative analysis. Voxel-based calculations and comparisons involving different MR volumes require estimation of the true noise-free signal to ensure reliable, unbiased inference. Also, registration algorithms often suffer from misleading information inherent in the noisy data [32].

Therefore, before the registration step, all analyzed images are submitted to 3-dimensional noise-removal routine. Denoising of MR magnitude images using standard image noise removal techniques is generally not fully justified, since thermal noise adds to raw data space and after its Fourier transform it has no more additive characteristics. It is best modeled by the Rician distribution, in fact. However, for higher SNR ratios (i.e. SNR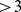) it can be well approximated by the Gaussian function [32], [33]. In the case of the available images, SNR ranges from around 16 to 23. Therefore, in this study we perform denoising of the real data assuming the Gaussian model of noise.

The noise removal procedure is accomplished using the non-local means (NLM) filter. Its exact definition is omitted here as it exceeds the scope of this paper. Instead, the reader is suggested to review references [34]–[36]. The principle of the algorithm operation consists in calculation of an output (denoised) pixel value based on the weighted mean of all other image pixels. Weights for a given pixel are determined by distances between this pixel neighborhood and neighborhoods of other image pixels. A neighborhood is defined as a set of all adjacent image points of a given pixel, excluding this pixel itself, while the distance is calculated as the L2 norm between relevant neighborhood pixel intensities. In our experiments we employ custom made implementation of the NLM algorithm extended to 3D.

#### Calculation results

The calculated correlation coefficients between simulated and real images are presented in Table 4. The upper and lower bounds for each coefficient assuming 95% confidence interval are also given. The correlation coefficients for Studies 1–3 range from 0.86 to 0.92 and for Study 4 the minimum correlation exists between 50%-stenosis tubes and equals to 0.78. The p-value remains below 0.001 in any case which proves validity of the designed simulation framework and its ability to synthesize realistic MRA images. It is observed that the largest local differences (exceeding the level of 30% of maximum brightness in the real image) appear near the vessel walls as well as in the region after the narrowing, where the main flux of the laminar flow deviates from long axis of the vessel. The calculated RMS error, however, falls into the range (11.5,16.8), below the value of standard deviation of both the denoised real images (45–61) and the synthetic ones (32–41).

**Table 4:** Correlation coefficients between simulated and real images.

Phantom	Study ID	Correlation coefficient	Lower bound	Upper bound	RMS error
U-bend	1	0.927	0.926	0.929	11.52
	2	0.927	0.925	0.929	11.68
	3	0.904	0.902	0.906	11.57
	4	0.811	0.809	0.814	13.24
50%-stenosis	1	0.918	0.919	0.921	12.01
	2	0.893	0.889	0.895	12.67
	3	0.911	0.909	0.912	11.89
	4	0.781	0.779	0.784	16.75
75%-stenosis	1	0.897	0.896	0.898	13.05
	2	0.881	0.879	0.884	13.52
	3	0.901	0.899	0.903	12.06
	4	0.875	0.877	0.879	13.97

#### Performance evaluation

The problem of computational efficiency of any complex and data-exhaustive system deserves individual and thorough analysis and it exceeds the scope of this work. Therefore, in this study, which is primarily concerned with the proposed MRA imaging model validation, we provide only partial evaluation of the execution performance. The following considerations refer solely to image synthesis, and the blood flow modeling, which precedes MRA-related routines, is not taken into account.

The MRA simulation time depends mostly on two factors – the k-space size (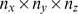) and the number of particles 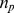. Table 5 presents simulation times measured for different values of these factors and for various grid configurations. The experiments were accomplished for the 50%-stenosis tube and – if not indicated otherwise – on the previously described computing grid (1 iMac and 10 MacMini computers).

**Table 5:** Performance eveluation for the 50%-stenosis tube.

Image size	Number of trajectories	Total number of particles	Number of processing slots	Using hyperthreading	Simulation time
128×128×25	128	71 967	48	Yes	6 m 15 s
		149 386	48	Yes	11 m 24 s
		298 651	48	Yes	23 m 56 s
192×192×44	256	202 141	48	Yes	53 m 59 s
192×192×44			24	No	1 h 5 m 53 s
128×128×44			48	Yes	23 m 39 s
128×128×25			48	Yes	13 m 14 s
128×128×25			24	No	15 m 52 s
128×128×25			16^a^	No	22 m 29 s
128×128×25			10^b^	No	36 m 25 s

a Grid composed of 1 iMac desktop and 6 MacMini units.

b Grid composed of 1 iMac desktop and 3 MacMini units.

The measured execution times span from single minutes to approximately 1 h for the most complicated case. These results show efficiency of the system implementation. The duration of a single image synthesis process allows for collecting of their relatively large database in a reasonable time period. This is crucial since large data sample is required for statistically significant evaluation of image processing algorithms.

A comparison of individual measurements reveals that the computational complexity is 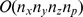. For example, experiments in rows 1–3 were performed on the same image size but various number of particles 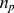. As it can be seen, the dependence on the execution time is linear. Similarly, the change on 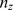 (measurements 6 and 7) in relation 1∶1.76 (25∶44) results in the equivalent increase of the simulation time (in seconds 794∶1419). Furthermore, it can be observed (rows 4 and 5) that using hyper-threading technology leads to improved efficiency, although this benefit is lower than the apparent increase in the number of parallel processing slots would suggest.

It must be underlined that the achieved high performance is in part guaranteed by the model itself. Image formation procedures involving the greatest computational burden iterate over particles i.e. actual sources of MR signal but not over every element of the imaged volume (potentially containing the air) as in the other simulators.

### Towards simulation of anatomical models

**Figure 15:**
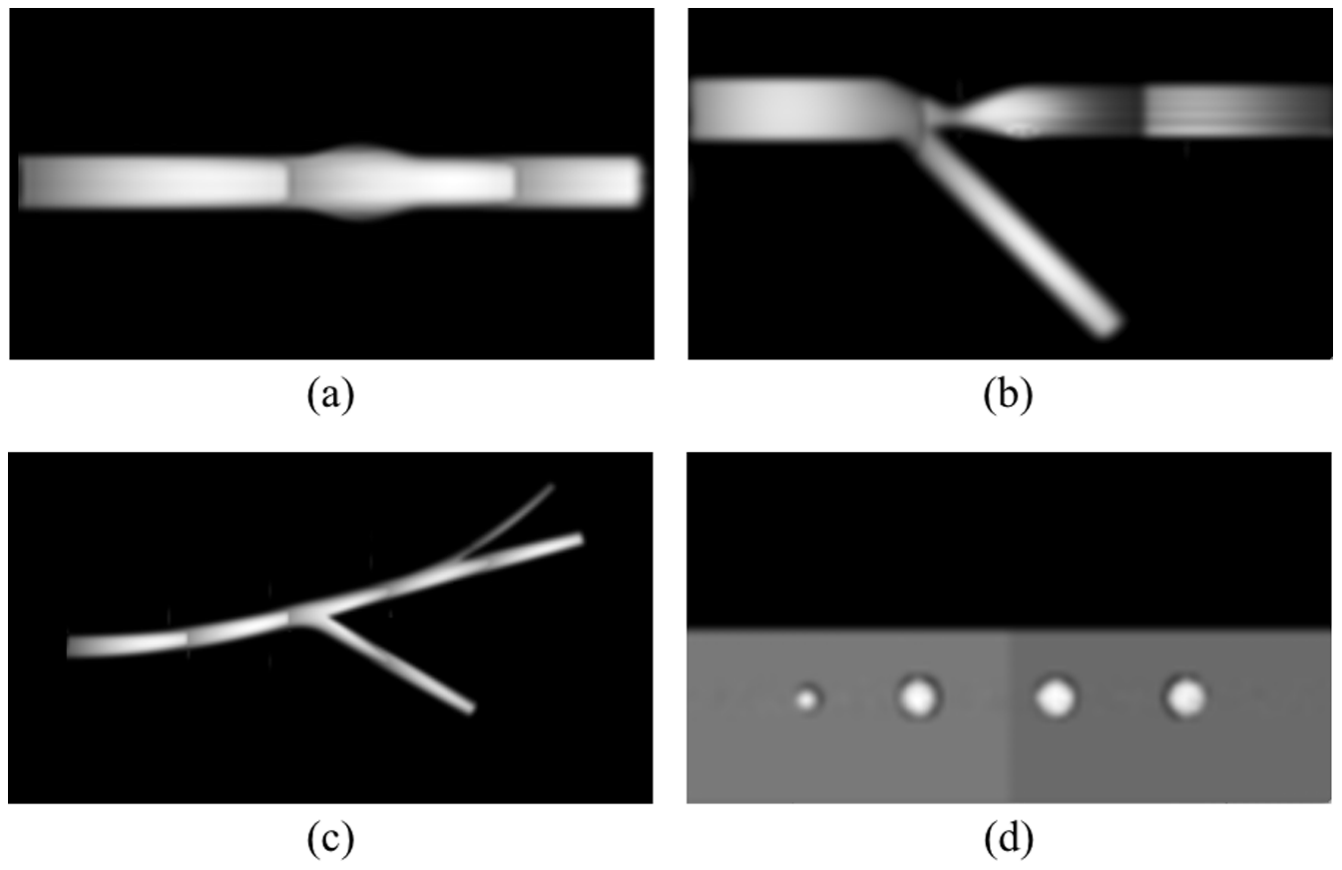
Sample images of realistic structures. A vessel with fusiform aneurysm (a), eccentric shape of stenosis at bifurcation (b) a vessel with side-branches (c) and stationary tissue around 4-vessel phantom (d). a–c) 3D acquisitions using both MOTSA and TONE options (TR/TE/FA = 27/4.6/15°), d) a 2D sequence (TR/TE/FA = 40/4.7/15°).

A comparative study presented above constitutes a formal approach to evaluate the designed system. The ultimate goal is to employ the simulator to collect a series of images for statistical analysis of vessel segmentation algorithms. Therefore, it is required that the produced images contain vessel geometries visible in actual MRA examinations. In this section we demonstrate capabilities of the technique in synthesizing images for structures anatomically more realistic than straight phantom tubes. In particular, Fig. 15a–c show the obtained images – coronal slices from 3D acquisitions – for a stenosed bifurcation, a vessel with fusiform aneurysm and a vessel with side-branches. The observed signal attenuation at stenosis and around aneurysm are characteristic for real MRA studies. These artefacts are caused in part by spin dephasing due to large local differences in flow velocity, especially near stenosis, and becuase of saturation of some molecules which slow down in the area of aneurysm. Note also the lower image intensity in a thinner side branch of a vessel in Fig. 15c. This intensity drop reflects the lower flow rate and signal averaging in a voxel covering both the edge and central axis of the vessel.

Finally, Fig. 15d presents a 2D image synthesized for a 4-tube digital phantom surrounded by two virtual tissue types. The stationary particles were assigned magnetic properties characteristic for gray and white matter. As expected, the signal produced by the moving spins is significantly enhanced relative to the stationary tissues.

## Discussion

The above presented comparisons constitute preliminary results of our research into the problem of designing versatile MRA simulation system. he obtained images contain many realistic details although some results fail to meet expectations. Most importantly this concerns the issue of too large intensity drops on the slab borders visible in images synthesized for the 10 ml/s flow rate (Fig. 13). This effect might be caused by an inappropriate adjustment of the excitation pulses. Real scanning systems implement various optimization procedures, which control – in the way hidden for the user – the inclination of a ramped flip angle. The exact dependence of FA from the 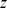 coordinate may not be strictly linear on the entire length of the slab. From the synthesized image it is clear that the range of the flip angle used in simulation (20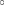 around nominal FA) has been too large to produce balanced signal for the whole slab. However, reducing this range would affect images of the other channels. Also, the U-bend phantom simulated in Study 4 (Fig. 11) deviates from its real counterpart, especially in the arc section. Undoubtedly, in case of larger flow rates, further research is needed into tuning of the simulation parameters in order to achieve closer resemblance to the true data acquisition procedures.

The Time-Of-Flight protocol in spite of its simplicity is quite demanding for computer modeling – both with respect to the underlying physical processes, as well as from the efficient implementation viewpoint. We approached the latter problem by decomposition of a single simulation experiment into a series of acquisitions, each concerned with its dedicated bundle of particle trajectories. Here, a very important advantage of our solution based on particle tracing becomes visible. A possible alternative approach to combined flow and MR simulation would utilize fixed lattice of nodes, where information about fluid flowing in and out is somehow stored and transferred to neighboring nodes. As for the distributed computing it would involve additional message passing between various computing grid agents. In our solution, the only communication events take place between the master and the agent computer and it happens at the beginning of simulation, when agent receives identifiers of trajectories to handle, and then after computations are done, when an agent sends the calculated signals to the master.

The designed system will be further developed to account for phenomena which are now disregarded. The problems which need to be addressed in the future can be grouped into two major issues corresponding to separate physical phenomena, i.e. blood flow simulation and MR physics. The first group of issues includes modeling of blood transport within a body part which is itself subject to motion. A blood particle position is then determined by two processes – hemodynamics and physiological displacement of an organ. It concerns important clinical applications of MRA such as diagnosis of cardiac anatomy, liver or kidney. Moreover, modeling of blood flow in the present study assumes fluid behavior which is either laminar or – in narrowed or bending regions – turbulent. Even in the latter case, however, it is a simplified model. The blood motion in such situations is chaotic and shear stress tensor should be estimated to account for non-Newtonian flow. Finally, more complex, anatomical topologies of the vessel system, as well as pulsatile flow will be considered in order to obtain more realistic MRA data sets.

Within the MRI part of the simulator, future efforts will be directed at including the effect of magnetization transfer used to maximize signal suppression from stationary tissue. Another issue that needs investigation is the consequence of using a special case analytic solution to the Bloch equation instead of a numerical solver. Redesigning the simulator to calculate exact solution of a differential equation will not only allow usage of time-varying gradient pulses, but also it will give an opportunity to quantitatively evaluate the degree of measurement error induced by analytical solution. The images synthesized in this study are noise-free. This assumption is justified in experiments where the goal is to solely verify the simulator operation. However, as far as the validation of MRA image processing algorithms is concerned, the simulation results should be corrupted by noise, so that the applied methods meet the same impediments characteristic for real images. Commonly the noise in MRI simulations is modeled as white Gaussian ergodic process added directly to the calculated k-space.

Nonetheless, the achieved results are satisfactory and meet the initial specification of system. The synthesized images contain much of information found in the real ones. The most important image features reproduced in the performed simulations are as follows:

- parabolic profile of the signal in regions of laminar flow,
- signal void on slab borders,
- specific flow patterns after stenoses,
- signal attenuation due to saturation, both in the stationary tissue and in fluid passing through a saturation band,
- higher signal on vessel walls than in the lumen in the channels with reverse flow direction.

In conclusion, these results encourage a follow-up study on quantitative validation of vessel segmentation algorithms applied to the MRA synthesized images.

## References

[1] Arlat I, Bongartz G, Marchal G (2002) Magnetic Resonance Angiography. Berlin: Springer Verlag.

[2] Bernstein MA, King KF, Zhou XJ (2004) Handbook of MRI Pulse Sequences. Amsterdam, The Netherlands: Academic Press.

[3] Haacke EM, Brown RW, Thompson MR, Venkatesan R (1999) Magnetic resonance imaging: physical principles and sequence design. New York: Wiley-Liss.

[4] Stamm AC, Wright CL, Knopp MV, Schmalbrock P, Heverhagen JT (2013) Phase contrast and time-of-ight magnetic resonance angiography of the intracerebral arteries at 1.5, 3 and 7 T. Magn Reson Imaging 31: 545–549. 23219250 10.1016/j.mri.2012.10.023

[5] Schneider G, Prince MR, Meaney JF, Ho VB (2005) Magnetic Resonance Angiography. Techniques, Indications and Practical Applications. Milan: Springer-Verlag Italia.

[6] Eiho S, Qian Y (1997) Detection of coronary artery tree using morphological operator. Proc Comput Cardiol 7: 525–528.

[7] Frangi AF, Niessen WJ, Vincken KL, Viergever MA (1998) Multiscale vessel enhancement filtering. In: Wells WM, Colchester A, Delp S, editors, Medical Image Computing and Computer-Assisted Interventation MICCAI'98, Springer Berlin Heidelberg, volume 1496 of *Lecture Notes in Computer Science*. pp. 130–137.

[8] Kirbas C, Quek F (2004) A review of vessel extraction techniques and algorithms. ACM Comput Surv 36: 81–121.

[9] Materka A, Strzelecki M, Szczypiński P, Kociński M, Deistung A, et al. (2009) Arteries tracking in simultaneous ToF-SWI MR images: image characteristics and preliminary results. In: Image and Signal Processing and Analysis, 2009. ISPA 2009. Proceedings of 6th International Symposium on. IEEE, pp. 748–753.

[10] Simmons A, Arridge SR, Barker GJ, Williams SC (1996) Simulation of MRI cluster plots and application to neurological applications. Magn Reson Imaging 14: 73–92. 8656992 10.1016/0730-725x(95)02040-z

[11] Petersson J, Christoffersson J, Golman K (1993) MRI simulation using the k-space formalism. Magn Reson Imaging 11: 557–568. 8316069 10.1016/0730-725x(93)90475-s

[12] Benoit-Cattin H, Collewet G, Belaroussi B, Saint-Jalmes H, Odet C (2005) The SIMRI project: a versatile and interactive MRI simulator. J Magn Reson Imaging 173: 97–115. 10.1016/j.jmr.2004.09.027 15705518

[13] Kwan RS, Evans A, Pike G (1999) MRI simulation-based evaluation of image-processing and classification methods. IEEE Trans Med Imaging 18: 1085–1097. 10661326 10.1109/42.816072

[14] Stoecker T, Vahedipour K, Pugfelder D, Shah NJ (2010) High-performance computing MRI simulations. Magn Reson Med 64: 186–193. 20577987 10.1002/mrm.22406

[15] Yoder DA, Zhao Y, Paschal CB, Fitzpatric JM (2004) MRI simulator with object-specific field map calculations. Magn Reson Imaging 22: 315–328. 15062927 10.1016/j.mri.2003.10.001

[16] Shelley Medical Imaging Technologies (2013) MRI Quality Assurance Flow Phantom Set. Available: http://www.simutec.com/. Accessed February 2014.

[17] Holdsworth DW, Rickey DW, Drangova M, Miller DJM, Fenster A (1991) Computer-controlled positive displacement pump for physiological ow simulation. Med Biol Eng Comput 29: 565–570. 1813750 10.1007/BF02446086

[18] Anderson JD (1995) Computational Fluid Dynamics. The Basics with Applications. New York: McGraw-Hill.

[19] Kwak D, Kiris CC (2011) Computation of Viscous Incompressible Flows. Springer.

[20] Wilcox DC (2006) Turbulence Modeling for CFD. DCW Industries.

[21] Hokr M, Maryska J (2005) Inuence of mesh geometry to numerical diffusion in upwind scheme for porous media solute transport. In: Proceedings of algoritmy. pp. 123–131.

[22] Holleman R, Fringer O, Stacey M (2013) Numerical diffusion for ow-aligned unstructured grids with application to estuarine modeling. Int J Numer Methods Fluids 72: 1117–1145.

[23] Comsol AB (2014) COMSOL Multiphysics Version 4.3, Particle Tracing User's Guide, Comsol AB, Stockholm, Sweden.

[24] Parker DL, Yuan C, Blatter DD (1991) MR angiography by multiple thin slab 3D acquisition. Magn Reson Med 17: 434–451. 2062215 10.1002/mrm.1910170215

[25] Carr J, Carroll T (2012) Magnetic Resonance Angiography: Principles and Applications. New York: Springer Science+Business Media.

[26] Kim SE, Parker DL (2012) Time-of-Flight Angiography,chapter 2. New York: Springer Science+Business Media. pp. 39–50.

[27] Liang ZP, Lauterbur PC (2000) Principles of Magnetic Resonance Imaging: A Signal Processing Perspective. New York: IEEE Press.

[28] The MathWorks,Inc. (2013) MATLAB and Signal Processing Toolbox Release 2013a, The MathWorks, Inc., Natick, Massachusetts, United States.

[29] Gabriel E, Fagg GE, Bosilca G, Angskun T, Dongarra JJ, et al. (2004) Open MPI: Goals, concept, and design of a next generation MPI implementation. In: Proceedings, 11th European PVM/MPI Users' Group Meeting. Budapest, Hungary, pp. 97–104.

[30] Klepaczko A, Szczypiński P, Dwojakowski G, Kociński M, Strzelecki M (2012) Computer simulation of magnetic resonance angiography imaging. Parallel implementation in heterogeneous computing environment. In: New Trends in Audio and Video Signal Processing: Algorithms, Architectures, Arrangements and Applications, NTAV/SPA 2012. Proceedings of the Joint Conference. pp. 43–48.

[31] The MathWorks, Inc. (2013) MATLAB and Image Processing Toolbox Release 2013a, The MathWorks, Inc., Natick, Massachusetts, United States.

[32] Coupé P, Manjón JV, Gedamu E, Arnold D, Robles M, et al (2010) Robust Rician noise estimation for MR images. Med Image Anal 14: 483–493. 20417148 10.1016/j.media.2010.03.001

[33] Nowak R (1999) Wavelet-based Rician noise removal for magnetic resonance imaging. IEEE Trans Image Process 8: 1408–1419. 18267412 10.1109/83.791966

[34] Buades A, Coll B, Morel JM (2005) A review of image denoising algorithms, with a new one. Multiscale Model Simul 4: 490–530.

[35] He L, Greenshields IR (2009) A Nonlocal Maximum Likelihood estimation method for Rician noise reduction in MR images. IEEE Trans Image Process 28: 165–172. 10.1109/TMI.2008.927338 19188105

[36] Zuo XN, Xing XX (2011) Effects of non-local diffusion on structural MRI preprocessing and default network mapping: Statistical comparisons with isotropic/anisotropic diffusion. PLoS One 6: e26703. 22066005 10.1371/journal.pone.0026703 PMC3204989

